# Association of interpregnancy interval with adverse pregnancy outcomes according to the outcomes of the preceding pregnancy: a longitudinal study with 4.7 million live births from Brazil

**DOI:** 10.1016/j.lana.2024.100687

**Published:** 2024-02-01

**Authors:** João Guilherme G. Tedde, Thiago Cerqueira-Silva, Sidney A. Lagrosa Garcia, Brenda V. Amira, Laura C. Rodrigues, Mauricio L. Barreto, Aline S. Rocha, Rita de Cássia Ribeiro-Silva, Ila R. Falcão, Enny S. Paixao

**Affiliations:** a School of Medicine, Federal University of Grande Dourados, Brazil; b Center of Data and Knowledge Integration for Health (CIDACS), Oswaldo Cruz Foundation, Salvador, Brazil; c Faculty of Medicine of São José do Rio Preto (FAMERP), Children's Hospital, São Paulo, Brazil; d School of Nutrition, Federal University of Bahia, Salvador, Brazil; e Faculty of Epidemiology and Population Health, London School of Hygiene and Tropical Medicine, London, UK

**Keywords:** Interpregnancy interval, Birth spacing, Perinatal outcomes, Preterm, Low birth weight, Small-for-gestational-age

## Abstract

**Background:**

Earlier studies have proposed a link between the Interpregnancy Interval (IPI) and unfavorable birth outcomes. However, it remains unclear if the outcomes of previous births could affect this relationship. We aimed to investigate whether the occurrence of adverse outcomes–small for gestational age (SGA), preterm birth (PTB), and low birth weight (LBW)–at the immediately preceding pregnancy could alter the association between IPI and the same outcomes at the subsequent pregnancy.

**Methods:**

We used a population-based linked cohort from Brazil (2001–2015). IPI was measured as the difference, in months, between the preceding birth and subsequent conception. Outcomes included SGA (<10th birthweight percentile for gestational age and sex), LBW (<2500 g), and PTB (gestational age <37 weeks). We calculated risk ratios (RRs), using the IPI of 18–22 months as the reference IPI category, we also stratified by the number of adverse birth outcomes at the preceding pregnancy.

**Findings:**

Among 4,788,279 births from 3,804,152 mothers, absolute risks for subsequent SGA, PTB, and LBW were higher for women with more adverse outcomes in the preceding delivery. The RR of SGA and LBW for IPIs <6 months were greater for women without previous adverse outcomes (SGA: 1.44 [95% Confidence Interval (CI): 1.41–1.46]; LBW: 1.49 [1.45–1.52]) compared to those with three previous adverse outcomes (SGA: 1.20 [1.10–1.29]; LBW: 1.24 [1.15–1.33]). IPIs ≥120 months were associated with greater increases in risk for LBW and PTB among women without previous birth outcomes (LBW: 1.59; [1.53–1.65]; PTB: 2.45 [2.39–2.52]) compared to women with three adverse outcomes at the index birth (LBW: 0.92 [0.78–1.06]; PTB: 1.66 [1.44–1.88]).

**Interpretation:**

Our study suggests that women with prior adverse outcomes may have higher risks for adverse birth outcomes in subsequent pregnancies. However, risk changes due to differences in IPI length seem to have a lesser impact compared to women without a prior event. Considering maternal obstetric history is essential in birth spacing counseling.

**Funding:**

10.13039/100010269Wellcome Trust225925/Z/22/Z.

**Research in context**

**Evidence before this study**

We searched PubMed on January, 2023 for English articles published using the search strategy: ("poor birth outcomes" OR "adverse birth outcomes" OR "preterm" OR ("low birth weight" OR "low weight") OR ("small for gestational age" OR "sga")) AND ("interpregnancy interval"[title] OR "birth spacing" [title]) AND ("previous" [title] OR "recurrence" [title] OR "history" [title]). We also included the reference lists of selected articles. The overall trend indicates that regardless of the length of the interpregnancy interval (IPI), mothers with previous conditions like preeclampsia, gestational diabetes, preterm birth, low birth weight, small-for-gestational-age, or stillbirth consistently face higher absolute risks for repeating these outcomes at the subsequent pregnancy. However, research findings have been inconsistent in determining the relative impact of prior outcomes on the association between IPI and subsequent outcomes. Most studies were conducted in high-income countries with limited sample sizes, and none have explored the cumulative impact of increasing numbers of adverse prior outcomes on the aforementioned association.

**Added value of this study**

This study represents the largest investigation into the influence of prior birth outcomes on the association between IPI and subsequent birth outcomes, particularly in a low- and middle-income country (LMIC) context, where the impact of such outcomes is of significant concern. By utilizing national data with extensive population coverage, we were able to assess the relative impact of IPI on the subsequent risk of SGA, LBW, and PTB, while considering the number of birth outcomes at the preceding pregnancy. We adjusted for several possible confounders and conducted sensitivity analyses for testing the robustness of our findings.

**Implications of all the available evidence**

Our findings reinforced the well described tendency for repeating the preceding outcome in the next pregnancy and that the association between IPI and subsequent perinatal outcomes may be influenced by prior occurrences, suggesting birth spacing recommendations should consider maternal obstetric history. Future research is needed to confirm our findings in populations with distinct baseline risks while controlling for clinically relevant maternal diseases and antenatal exposures.

## Introduction

Available evidence shows that having a small for gestational age (SGA), preterm (PTB), or low birth weight (LBW) child increases the risk of recurrence in subsequent pregnancies.^1^, ^2^, ^3^, ^4^ These adverse perinatal outcomes have significant clinical implications, as affected infants face higher neonatal morbidity, mortality, and long-term health issues.^5^, ^6^, ^7^ Interpregnancy interval (IPI), the time between delivery and conception in the next pregnancy, is recognized as a potentially modifiable risk factor for adverse perinatal outcomes, including SGA, PTB, and LBW, with both short and long IPIs linked to elevated risks.^8^, ^9^, ^10^, ^11^, ^12^, ^13^, ^14^ Measures such as family planning and use of contraceptive methods can modify this risk factor, being included in the World Health Organization (WHO) recommendation on birth spacing,^15^ which suggests waiting at least 24 months after a live birth and 6 months after a miscarriage or induced abortion before trying to conceive again.

However, it remains unclear whether IPI length affects adverse birth outcomes similarly in women with a history of prior adverse perinatal outcomes compared to those without such history. Some studies^16^, ^17^, ^18^, ^19^, ^20^, ^21^ have suggested a potential change in this association for different maternal and infant outcomes depending on the maternal history of prior adverse outcomes. However, most of these studies were conducted in high-income countries with small sample sizes, which raises concerns about the applicability of their results to low- and middle-income countries, where healthcare access, maternal exposures, and disease profiles may vary significantly. Moreover, to date, no study has assessed the recurrence risks of PTB, SGA and LBW based on the number of prior adverse outcomes. In this analysis we aim to investigate whether the association between interpregnancy interval length and pregnancy outcomes vary by the presence of prior adverse perinatal outcomes, using data from the Centre for Data and Knowledge Integration for Health (CIDACS) Birth Cohort.

## Methods

### Study population

We used the CIDACS Birth Cohort–a linked dataset combining the national live birth system of Brazil (Sistema de Informação sobre Nascimentos [SINASC]^22^) and the 100 million Brazilian Cohort baseline^23^–for the period between January 1, 2001 and December 31, 2015. It is composed of 24,695,617 live births that, in general, were born from younger, unmarried and less educated mothers.^24^ The linkage was performed at CIDACS in a strict data protection environment and according to ethical and legal regulations,^25^ using CIDACS RL-Record Linkage, a tool developed to link large-scale administrative datasets.^26^ Detailed information on data sources and linkage accuracy have been reported previously^27^ and can be found in the Supplementary Material (Additional Methods).

Our study included eligible women with at least two consecutive, singleton live births. To limit each woman's contribution, we excluded births of sixth or higher order, allowing a maximum of 5 children per woman (99th percentile in our cohort). We also excluded births with gestational ages outside the 22–43 weeks range based on reference growth charts.^28^ Additionally, mothers younger than 14 or older than 50 years were excluded, along with births with birthweights below 500 g or above 6000 g, as these values are associated with a non-viability or extremely low likelihood of occurrence. Finally, we limited our analysis to IPIs of one month or longer, as the available data on postpartum fertility suggests that conceptions occurring earlier than this are highly improbable.^29^

**Fig. 1:**
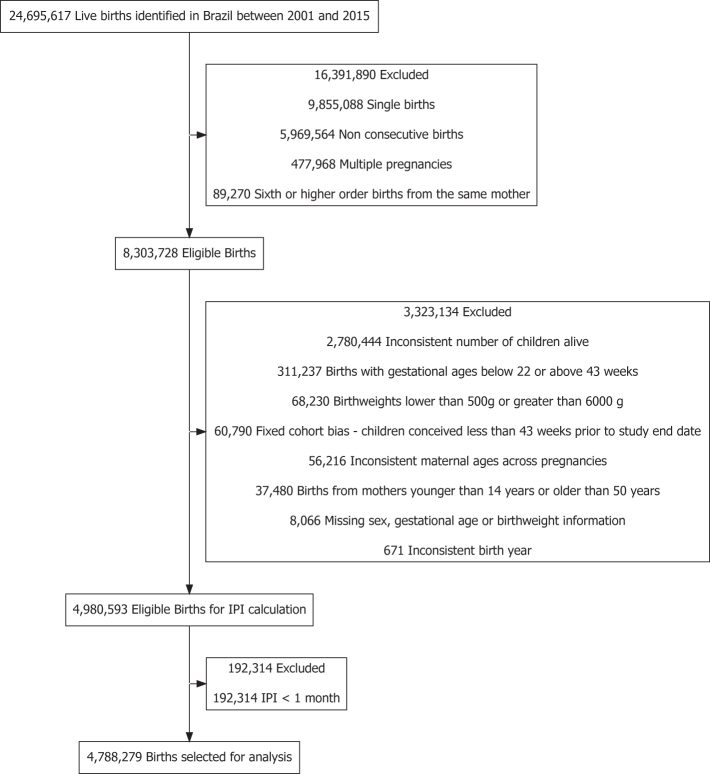
**Flowchart of studypopulation.**

To reduce fixed cohort bias,^30^^,^^31^ we restricted the cohort to children with a conception date up to 43 weeks before the cohort final date (December 31, 2015). Women with more than one eligible interpregnancy intervals in the study period could contribute more than one observation for the analyses. Fig. 1 provides additional information on study inclusion criteria.

Ethical approval was obtained from the Federal University of Bahia’s Institute of Public Health Ethics Committee (CAAE registration number: 18022319.4.0000.5030).

### Exposure and outcome definitions

Interpregnancy interval was defined as the time in months between an index birth (i.e., the first birth among two consecutive births) and the subsequent conception for a given mother. It was calculated by subtracting the index birth date from the subsequent birth date minus the gestational age at birth of the subsequent pregnancy (See Additional Methods and Supplementary Figure S1—Supplementary Material).

For example, given a woman with 3 consecutive deliveries, IPI 1 = (conception date of child 2–delivery of child 1) → (delivery of child 2–gestational age of child 2)–delivery of child 1 → (December, 15, 2005—36 weeks)–December, 15, 2001 ≃ 39 months. IPI 2 = (conception date of child 3–delivery of child 2) → (delivery of child 3–gestational age of child 3)–delivery of child 2 → (December, 15, 2010—36 weeks)–December, 15, 2005 ≃ 51 months. We treated IPI as categorical, (<6, 6–10, 11–17, 18–22, 23–58, 59–119, ≥120 months), to allow for comparisons with previous studies.

Given the changes in gestational age registration in our dataset over time, specifically from non-overlapping categories of completed weeks to exact discrete values starting from 2011, we employed an algorithm for imputing precise gestational age values within the original range of possible values for births that occurred prior to 2011 (see Additional Methods in the Supplementary Material for details and code used).

Outcomes included: (1) SGA (birth weight <10th percentile for sex and gestational age based on intergrowth charts),^28^ (2) PTB (delivery at less than 37 weeks) and (3) LBW (birth weight <2500 g).

### Statistical analysis

We examined the association between interpregnancy interval and each outcome at the subsequent pregnancy, first in the overall population, and then stratified by the number of adverse outcomes (SGA, PTB, and LBW) at the index birth. For each subsequent outcome, we fit a logistic regression model adjusted for baseline covariates (i.e., measured at index birth) considered potential confounders of the association between IPI and adverse birth outcomes (See Supplementary Figures S1 and S2). Covariates included the presence of any congenital anomaly, mother’s age, number of antenatal visits, maternal education, region of birth, mode of delivery, number of live children and birth year.

To investigate whether adverse outcomes at the index birth influenced the association between IPI and subsequent adverse outcomes, we introduced a multiplicative interaction term involving the IPI category and the number of adverse outcomes at the index birth.

Absolute risks were estimated from the logistic regression model. The predicted log-odds were employed to calculate probabilities, which were used to compute risk ratios (RR) with 95% Confidence Intervals (CIs). Standard errors were calculated using the delta method.^32^

We estimated adjusted risks first for the overall population, stratified only for IPI categories, then for IPI category and number of index birth outcomes. We used robust variance estimation to account for the non-independence of 2 or more interpregnancy intervals belonging to the same woman.

Finally, we estimated RRs comparing risks at each IPI category with predicted risks at the 18–22-month length (reference category), first for the overall population and then stratified by number of index birth outcomes. Findings were considered significant if the 95% CI did not cross the reference value 1.

In a *post-hoc* exploratory analysis, we replicated the models used in the initial analysis, but this time, we employed Large-for-Gestational-Age (LGA–birth weight >90th percentile for sex and gestational age based on intergrowth charts) as the primary outcome for the subsequent birth. Our aim was to examine whether the risks associated with LGA births showed different patterns when compared to SGA births concerning Interpregnancy Interval. This exploration was motivated by the possibility that, as IPI lengthens, unique risk profiles, such as gestational diabetes and obesity, may favour LGA over SGA births.

All IPIs with missing values on covariates were excluded from the main analysis, with 4,422,146 intervals included in the regression models. All analyses were performed in R statistical software (version 3.6.0).^33^ See Additional Methods (Supplementary Material) for details on statistical analysis.

### Sensitivity analyses

To see if our results vary by the type of outcome at the index birth, we fit another model using the same covariates of the primary analysis, but instead of representing index birth outcome history by the number of previous outcomes (0–3), we separated into 7 categories, each one accounting for a possible outcome (none, SGA, PTB, LBW, SGA and LBW, PTB and LBW, SGA and LBW and PTB). We also conducted additional analyses to assess the robustness of our findings. We (1) restricted analysis to children born from 2011 onwards to test if gestational age imputation may have biased the results; (2) used the outcome of the index pregnancy as a negative control, expecting to see no association between the outcome of the index birth and interpregnancy interval, and therefore, no residual unmeasured confounding. These approaches are detailed in the Supplementary Material (Additional Methods).

### Role of the funding source

The funder of the study had no role in study design, data collection, data analysis, data interpretation, or writing of the report. The corresponding author had full access to all the data in the study and had final responsibility for the decision to submit for publication.

## Results

Our final dataset included 3,804,152 women and 4,788,279 intervals. Of these, 1,871,449 (39.1%) had length within 23–58 months, while 268,006 (5.6%) and 77,462 (1.6%) had extreme lengths (i.e., <6 months and ≥120 months, respectively). Most intervals (3,464,102 [72.3%]), represented mother's first eligible intervals, while 46,639 (1.0%) corresponded to fourth intervals. Moreover, 4,401,930 (68.8%) of them occurred after deliveries by mothers under 24 years old at the time of index birth. Additionally, 4,493,957 (95.1%) intervals were associated with women who had up to 11 years of education, while 3,089,675 (65.5%) were attributed to non-married women (including those who were single, divorced, or widowed).

Index births combining three adverse outcomes were more commonly followed by extremely short IPIs (<6 months) compared to those without adverse outcomes (10.4% vs 5.3%). Also, they occurred more often in very young (<18 years old) mothers (25.2% vs 18.0%), in South East region (43.0% vs 34.9%), had higher frequencies of pelvic, podalic or transverse presentations (10.9% vs 2.4%), were more likely to be delivered via cesarean delivery (42.3% vs 30.6%) and had higher prevalence of congenital anomaly detected at the time of delivery (3.6% vs 0.5%). In addition, in the group with the highest number of adverse outcomes at index birth, we observed a greater prevalence of inadequate prenatal care (i.e., <7 antenatal visits), with only 23.1% reaching the recommended number of visits compared to 48.3% in the group with no adverse outcomes. However, this rate increased significantly in the subsequent pregnancy, especially for women with 3 previous adverse outcomes, reaching 48.8%. The characteristics of the study population stratified by the number of adverse outcomes at the index birth are reported in Table 1.

**Table 1:** Cohort characteristics according to the number adverse outcomes at index birth.

Variables	Overall (N = 4,788,279)	Number of outcomes at index birth			
		0 (N = 3,943,890)	1 (N = 503,629)	2 (N = 298,411)	3 (N = 42,349)
**IPI (months)**					
<6	268,006 (5.6%)	207,188 (5.3%)	31,993 (6.4%)	24,409 (8.2%)	4416 (10.4%)
6–10	476,045 (9.9%)	381,226 (9.7%)	54,839 (10.9%)	35,012 (11.7%)	4968 (11.7%)
11–17	763,466 (15.9%)	622,049 (15.8%)	86,103 (17.1%)	48,807 (16.4%)	6507 (15.4%)
18–22	467,521 (9.8%)	383,659 (9.7%)	51,714 (10.3%)	28,499 (9.6%)	3649 (8.6%)
23–58	1,871,449 (39.1%)	1,555,840 (39.4%)	190,291 (37.8%)	110,194 (36.9%)	15,124 (35.7%)
59–119	864,330 (18.1%)	728,886 (18.5%)	81,235 (16.1%)	47,194 (15.8%)	7015 (16.6%)
≥120	77,462 (1.6%)	65,042 (1.6%)	7454 (1.5%)	4296 (1.4%)	670 (1.6%)
**Interval rank**					
1	3,464,102 (72.3%)	2,830,376 (71.8%)	372,798 (74.0%)	227,736 (76.3%)	33,192 (78.4%)
2	1,045,561 (21.8%)	881,105 (22.3%)	101,943 (20.2%)	55,205 (18.5%)	7308 (17.3%)
3	231,977 (4.8%)	193,946 (4.9%)	23,767 (4.7%)	12,731 (4.3%)	1533 (3.6%)
4	46,639 (1.0%)	38,463 (1.0%)	5121 (1.0%)	2739 (0.9%)	316 (0.7%)
**Index birth year**					
2001–2004	1,698,924 (35.5%)	1,412,839 (35.8%)	170,422 (33.8%)	100,778 (33.8%)	14,885 (35.1%)
2005–2010	2,375,294 (49.6%)	1,975,031 (50.1%)	232,369 (46.1%)	145,887 (48.9%)	22,007 (52.0%)
2011–2015	714,061 (14.9%)	556,020 (14.1%)	100,838 (20.0%)	51,746 (17.3%)	5457 (12.9%)
**Region of birth**					
South east	1,701,847 (35.5%)	1,375,701 (34.9%)	187,900 (37.3%)	120,019 (40.2%)	18,227 (43.0%)
South	631,846 (13.2%)	517,106 (13.1%)	66,274 (13.2%)	42,132 (14.1%)	6334 (15.0%)
Midwest	372,411 (7.8%)	308,357 (7.8%)	39,136 (7.8%)	21,973 (7.4%)	2945 (7.0%)
North east	1,530,705 (32.0%)	1,282,369 (32.5%)	151,831 (30.1%)	84,855 (28.4%)	11,650 (27.5%)
North	551,470 (11.5%)	460,357 (11.7%)	58,488 (11.6%)	29,432 (9.9%)	3193 (7.5%)
**Sex of index child**					
Male	2,465,936 (51.5%)	2,027,751 (51.4%)	279,417 (55.5%)	137,117 (45.9%)	21,651 (51.1%)
Female	2,322,343 (48.5%)	1,916,139 (48.6%)	224,212 (44.5%)	161,294 (54.1%)	20,698 (48.9%)
**Sex of subsequent child**					
Male	2,452,495 (51.2%)	2,020,944 (51.2%)	257,570 (51.1%)	152,375 (51.1%)	21,606 (51.0%)
Female	2,335,784 (48.8%)	1,922,946 (48.8%)	246,059 (48.9%)	146,036 (48.9%)	20,743 (49.0%)
**Child ethnicity at index birth**^a^					
White	1,441,123 (32.2%)	1,190,661 (32.3%)	146,810 (31.2%)	90,155 (32.3%)	13,497 (34.1%)
Black	422,072 (9.4%)	340,644 (9.2%)	47,423 (10.1%)	29,706 (10.7%)	4299 (10.9%)
Asian	18,859 (0.4%)	15,560 (0.4%)	2013 (0.4%)	1107 (0.4%)	179 (0.5%)
Brown	2,565,003 (57.3%)	2,117,639 (57.4%)	270,069 (57.4%)	155,943 (55.9%)	21,352 (54.0%)
Indigenous	33,172 (0.7%)	26,539 (0.7%)	4483 (1.0%)	1954 (0.7%)	196 (0.5%)
**Maternal age at index birth (years)**					
<18	907,300 (18.9%)	710,010 (18.0%)	113,619 (22.6%)	73,006 (24.5%)	10,665 (25.2%)
18–23	2,390,575 (49.9%)	1,980,061 (50.2%)	249,772 (49.6%)	141,796 (47.5%)	18,946 (44.7%)
24–29	1,104,055 (23.1%)	933,714 (23.7%)	103,042 (20.5%)	58,704 (19.7%)	8595 (20.3%)
30–34	306,815 (6.4%)	255,908 (6.5%)	28,933 (5.7%)	18,911 (6.3%)	3063 (7.2%)
35–39	73,870 (1.5%)	59,785 (1.5%)	7638 (1.5%)	5473 (1.8%)	974 (2.3%)
40–50	5664 (0.1%)	4412 (0.1%)	625 (0.1%)	521 (0.2%)	106 (0.3%)
**Maternal education**^b^					
None	65,282 (1.4%)	53,756 (1.4%)	7108 (1.4%)	3955 (1.3%)	463 (1.1%)
1–3 years	425,422 (9.0%)	352,386 (9.1%)	44,600 (9.0%)	25,539 (8.7%)	2897 (6.9%)
4–7 years	1,800,348 (38.1%)	1,474,098 (37.9%)	195,277 (39.3%)	115,544 (39.2%)	15,429 (36.9%)
8–11 years	2,202,905 (46.6%)	1,817,141 (46.7%)	228,192 (46.0%)	136,754 (46.4%)	20,818 (49.8%)
≥12 years	228,704 (4.8%)	192,497 (4.9%)	21,364 (4.3%)	12,631 (4.3%)	2212 (5.3%)
**Maternal marital status at index birth**^c^					
Married or civil union	1,629,785 (34.5%)	1,358,899 (35.0%)	165,847 (33.4%)	92,136 (31.3%)	12,903 (30.9%)
Single, divorced or widowed	3,089,675 (65.5%)	2,528,234 (65.0%)	330,443 (66.6%)	202,130 (68.7%)	28,868 (69.1%)
Cesarean delivery at index birth^d^	1,452,915 (30.4%)	1,204,601 (30.6%)	139,168 (27.7%)	91,227 (30.6%)	17,919 (42.3%)
Cesarean delivery at subsequent birth^e^	1,859,634 (38.9%)	1,565,727 (39.7%)	171,374 (34.1%)	104,273 (35.0%)	18,260 (43.2%)
**Number of antenatal visits at index birth**^f^					
None	126,081 (2.7%)	93,776 (2.4%)	15,749 (3.2%)	13,794 (4.7%)	2762 (6.7%)
1–3	544,285 (11.5%)	411,282 (10.5%)	69,312 (13.9%)	53,751 (18.3%)	9940 (24.0%)
4–6	1,859,758 (39.3%)	1,511,142 (38.7%)	204,164 (41.0%)	125,221 (42.6%)	19,231 (46.3%)
≥7	2,206,089 (46.6%)	1,886,952 (48.3%)	208,454 (41.9%)	101,115 (34.4%)	9568 (23.1%)
**Number of antenatal visits at subsequent birth**^g^					
None	119,774 (2.5%)	92,996 (2.4%)	14,925 (3.0%)	10,434 (3.5%)	1419 (3.4%)
1–3	530,202 (11.2%)	419,839 (10.7%)	64,791 (13.0%)	40,324 (13.7%)	5248 (12.5%)
4–6	1,715,193 (36.1%)	1,408,312 (36.0%)	184,270 (36.9%)	107,860 (36.5%)	14,751 (35.2%)
≥7	2,382,016 (50.2%)	1,990,206 (50.9%)	234,786 (47.1%)	136,590 (46.3%)	20,434 (48.8%)
Anomaly at the index birth^h^	29,853 (0.7%)	18,974 (0.5%)	4114 (0.9%)	5334 (1.9%)	1431 (3.6%)
Anomaly at the subsequent birth^i^	31,703 (0.7%)	25,663 (0.7%)	3331 (0.7%)	2353 (0.8%)	356 (0.9%)
SGA at index birth	565,157 (11.8%)	0 (0.0%)	341,898 (67.9%)	180,910 (60.6%)	42,349 (100.0%)
SGA at subsequent birth	404,296 (8.4%)	257,038 (6.5%)	72,715 (14.4%)	65,223 (21.9%)	9320 (22.0%)
LBW at index birth	351,850 (7.3%)	0 (0.0%)	11,090 (2.2%)	298,411 (100.0%)	42,349 (100.0%)
LBW at subsequent birth	281,801 (5.9%)	161,834 (4.1%)	48,934 (9.7%)	59,938 (20.1%)	11,095 (26.2%)
Preterm at index birth	310,491 (6.5%)	0 (0.0%)	150,641 (29.9%)	117,501 (39.4%)	42,349 (100.0%)
Preterm at subsequent birth	358,981 (7.5%)	245,756 (6.2%)	54,234 (10.8%)	49,340 (16.5%)	9651 (22.8%)

Values are expressed as N (%). Number of outcomes at index birth: 0 (neither SGA, PTB or LBW); 1 (either SGA, LBW or PTB); 2 (SGA-LBW, PTB-LBW); 3 (SGA-LBW-PTB).

IPI: interpregnancy interval; SGA: small for gestational age; LBW: low birth weight; PTB: preterm birth.

a Missing = 308,050 (6.4%).

b Missing = 65,618 (1.4%).

c Missing = 68,819 (1.4%).

d Missing = 5565 (0.1%).

e Missing = 5221 (0.1%).

f Missing = 52,066 (1.1%).

g Missing = 41,094 (0.9%).

h Missing = 257,257 (5.4%).

i Missing = 163,837 (3.4%).

The prevalence of subsequent SGA, LBW, and PTB in the overall population was 8.4%, 5.9%, and 7.5%, respectively. For all the outcomes (SGA, LBW and PTB), their incidence in the subsequent pregnancy was proportionally raised as the number of adverse outcomes at the index pregnancy increased from 0 to 3. Supplementary Table S1 (Supplementary Material) displays the proportions of each type of outcome according to the number of adverse outcomes at index birth.

**Fig. 2:**
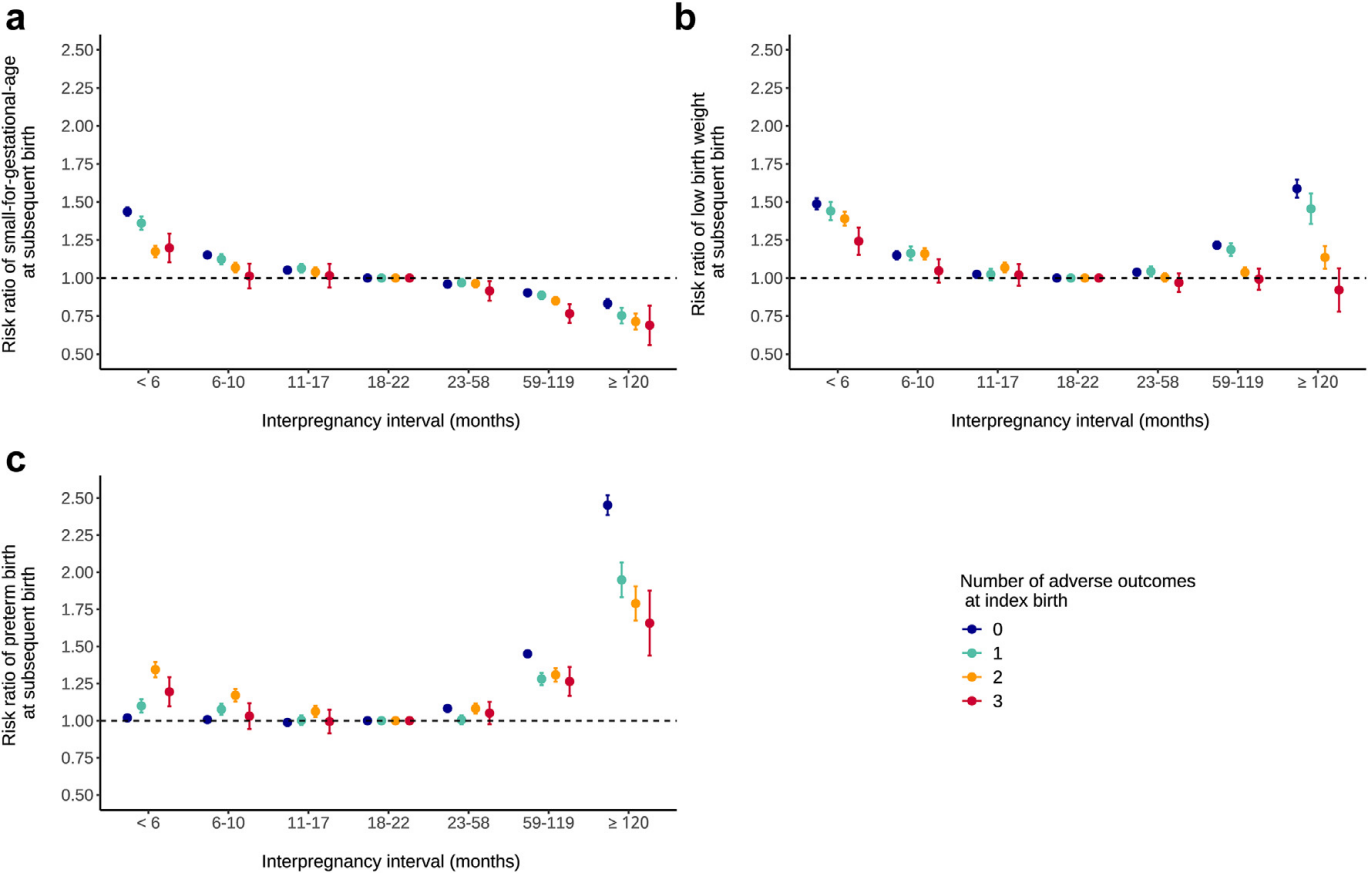
**Absolute risks of birth outcomes according to interpregnancy interval and number of adverse outcomes at index birth, Brazil, 2001–2015 in 4,422,146 pregnancies.** Legend: adjusted predicted risks (95% confidence intervals) of small-for-gestational-age, low birth weight and preterm birth at each interpregnancy interval length and according to the number of adverse outcomes at the index birth.

**Fig. 3:**
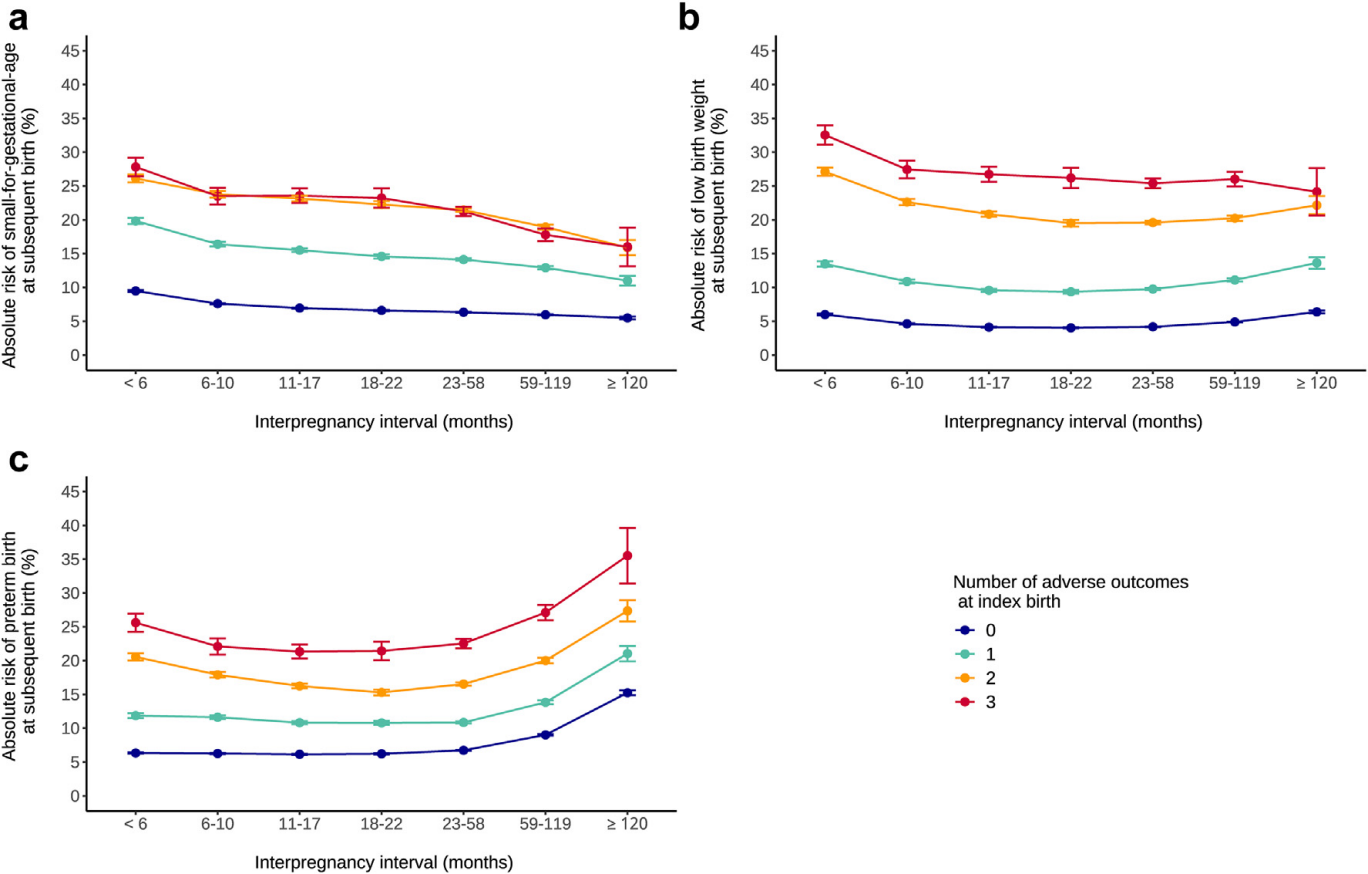
**Risk ratios of birth outcomes according to interpregnancy interval and number of adverse outcomes at index birth, Brazil, 2001–2015 in 4,422,146 pregnancies.** Legend: adjusted risk ratios (95% confidence intervals) of small-for-gestational-age, low birth weight and preterm birth at each interpregnancy interval length and according to the number of adverse outcomes at the index birth.

Adjusted absolute risks (Fig. 2 and Supplementary Table S2 [Supplementary Material]) and risk ratios (Fig. 3 and Table 2) for SGA, LBW, and PTB across IPI categories are presented for the overall study population and stratified by the number of index birth outcomes. In unstratified analysis, interpregnancy intervals <6 months and ≥120 months were associated with increases in risk of PTB (predicted risk, 8.4% for IPI <6 months and 7.1% for 18–22 months; RR, 1.18; 95% CI, 1.16–1.20 and 10.7% for IPI ≥120 months; RR, 1.49; 95% CI, 1.46–1.53) and LBW (predicted risk, 8.7% for IPI <6 months and 5.3% for 18–22 months; RR, 1.64; 95% CI, 1.61–1.67 and 7.3% for IPI ≥120 months; RR, 1.38; 95% CI, 1.34–1.42). For SGA, only very short IPIs were associated with increased risks in the overall population (predicted risk, 12.0% for IPI <6 months and 8.2% for 18–22 months; RR, 1.47; 95% CI, 1.45–1.49), while long IPIs were associated with decreased risk of SGA (predicted risk, 7.2% for IPI ≥120 months; RR 0.89; 95% CI, 0.86–0.91). In the post-hoc exploratory analysis (Supplementary Figure S3–Supplementary Material), after stratification for the number of index birth outcomes, LGA at the subsequent birth showed an overall increase in risks after longer IPIs, whereas IPIs shorter than 6 months were associated with reduced risks, which contrasted with the observed pattern for SGA.

**Table 2:** Risk ratios of each outcome (SGA, PTB, LBW) for IPI categories, stratified by number of index birth outcomes, compared with the reference level (18–22 months).

Outcome	Interpregnancy interval, aRR (95% CI)						
	<6	6–10	11–17	18–22	23–58	59–119	≥120
**SGA at subsequent birth**							
Overall	1.47 (1.45–1.49)	1.16 (1.14–1.17)	1.06 (1.04–1.07)	(Ref.)	0.97 (0.96–0.98)	0.92 (0.91–0.93)	0.89 (0.86–0.91)
**Previous outcomes at index birth (N)**							
0	1.44 (1.41–1.46)	1.15 (1.13–1.17)	1.05 (1.03–1.07)	(Ref.)	0.96 (0.95–0.97)	0.90 (0.89–0.92)	0.83 (0.80–0.86)
1	1.36 (1.32–1.40)	1.12 (1.09–1.16)	1.06 (1.03–1.09)	(Ref.)	0.97 (0.94–0.99)	0.89 (0.86–0.91)	0.75 (0.70–0.81)
2	1.17 (1.14–1.21)	1.07 (1.04–1.10)	1.04 (1.01–1.07)	(Ref.)	0.96 (0.94–0.99)	0.85 (0.82–0.88)	0.71 (0.66–0.77)
3	1.20 (1.10–1.29)	1.01 (0.93–1.09)	1.01 (0.94–1.09)	(Ref.)	0.91 (0.85–0.98)	0.77 (0.70–0.83)	0.69 (0.56–0.82)
**Low birth weight at subsequent birth**							
Overall	1.64 (1.61–1.67)	1.21 (1.19–1.23)	1.04 (1.03–1.06)	(Ref.)	1.01 (1.00–1.03)	1.12 (1.10–1.14)	1.38 (1.34–1.42)
**Previous outcomes at index birth (N)**							
0	1.49 (1.45–1.52)	1.15 (1.12–1.17)	1.02 (1.00–1.05)	(ref.)	1.04 (1.02–1.06)	1.22 (1.19–1.24)	1.59 (1.53–1.65)
1	1.44 (1.38–1.50)	1.16 (1.12–1.21)	1.02 (0.99–1.06)	(Ref.)	1.04 (1.01–1.08)	1.19 (1.14–1.23)	1.46 (1.36–1.56)
2	1.39 (1.34–1.43)	1.16 (1.12–1.20)	1.07 (1.03–1.10)	(Ref.)	1.00 (0.98–1.03)	1.04 (1.00–1.07)	1.14 (1.06–1.21)
3	1.24 (1.15–1.33)	1.05 (0.97–1.12)	1.02 (0.95–1.09)	(Ref.)	0.97 (0.91–1.03)	1.00 (0.92–1.06)	0.92 (0.78–1.06)
**Preterm birth at subsequent birth**							
Overall	1.18 (1.16–1.20)	1.09 (1.07–1.10)	1.02 (1.00–1.03)	(Ref.)	0.99 (0.98–1.00)	1.14 (1.13–1.16)	1.49 (1.46–1.53)
**Previous outcomes at index birth (N)**							
0	1.02 (1.00–1.04)	1.00 (0.98–1.02)	0.99 (0.98–1.00)	(Ref.)	1.08 (1.06–1.09)	1.45 (1.42–1.47)	2.45 (2.39–2.52)
1	1.10 (1.05–1.14)	1.07 (1.03–1.11)	1.00 (0.97–1.03)	(Ref.)	1.00 (0.97–1.03)	1.28 (1.24–1.32)	1.95 (1.83–2.07)
2	1.34 (1.29–1.39)	1.17 (1.12–1.21)	1.06 (1.02–1.10)	(Ref.)	1.08 (1.04–1.11)	1.30 (1.26–1.35)	1.79 (1.67–1.90)
3	1.20 (1.10–1.29)	1.03 (0.94–1.11)	0.99 (0.91–1.07)	(Ref.)	1.5 (0.98–1.12)	1.26 (1.16–1.36)	1.66 (1.44–1.88)

Data are presented as adjusted risk ratios (lower 95% CI—Upper 95% CI). Number of outcomes at index birth: 0 (neither SGA, PTB or LBW); 1 (either SGA, LBW or PTB); 2 (SGA-LBW, PTB-LBW); 3 (SGA-LBW-PTB).

IPI: interpregnancy interval; SGA: small for gestational age; LBW: low birth weight; PTB: preterm birth; Index birth: The first birth among two consecutive births from the same woman.

We found similar results after stratification by the number of index birth outcomes. For all IPI categories, absolute risks of a subsequent adverse outcome increased as the number of previous outcomes rose (Fig. 2 and Supplementary Table S2). Risks of SGA, LBW and PTB ranged respectively from 5.5% to 9.5%, 4.0% to 6.4% and 6.1% to 15.2% for the group without any adverse outcome at index birth, and 16.0%–27.8%, 24.1%–32.5% and 21.1%–35.5% for the group with 3 previous outcomes. However, regarding risk ratios, we observed the opposite trend, with values generally decreasing as the number of index birth outcomes increased. An exception was observed in the prematurity analysis, where the increased risk at short interpregnancy intervals was more pronounced in the group with more previous adverse outcomes (RR, 1.34; 95% CI, 1.29–1.39 for women with two previous outcomes compared to RR of 1.02; 95% CI, 1.00–1.04 for women with none). For all three outcomes, the interaction between IPI and the number of index birth outcomes was statistically significant (p < 0.001).

### Sensitivity analyses

After stratifying by the type of index birth outcome (Supplementary Table S3—Supplementary Material), results did not change meaningfully, with a general trend for higher risk ratios after extreme IPIs (i.e., <6 months and ≥120 months) and among women without previous adverse outcomes. An exception was noted again for the risk of SGA, which decreased as IPIs increased, a finding consistent with the primary analysis.

In the analysis restricted to 2011–2015 (Supplementary Figure S4—Supplementary Material), similarly to our main findings, we observed greater absolute risks following indexes births with the highest numbers of adverse outcomes. Nevertheless, when examining risk ratios (Supplementary Figure S5—Supplementary Material), except for preterm analysis, we noticed a distinct trend compared to the primary analysis, with higher point estimates in the groups with more index birth outcomes. However, due to overlapping 95% confidence intervals, the interpretation of these findings became challenging. Additionally, our examination was limited to IPIs not exceeding 50 months, representing the maximum observed length within the restricted cohort's period. Finally, using the outcome of the index pregnancy as a negative control (Supplementary Figure S6–Supplementary Material) produced different curve shapes, with none or attenuated relationships with IPI length, thus providing evidence against residual confounding.

## Discussion

The study found increased risks of SGA, LBW, and PTB after IPIs <6 months. IPIs ≥120 months were associated with increased risks only for PTB and LBW. These trends persisted after stratification by the number of adverse outcomes at the index birth. Regardless of IPI, absolute risks for subsequent SGA, LBW, or PTB were greater for women with previous adverse outcomes. However, risk ratios generally decreased as the number of adverse outcomes at the index birth increased, except for PTB after IPIs <6 months, where we found higher risk ratios in the group with more previous adverse outcomes.

Our findings align with previous studies assessing various perinatal outcomes,^17^, ^18^, ^19^, ^20^, ^21^^,^^34^ confirming the J-shaped relationship between IPI and birth outcomes and reinforcing the increased likelihood of repeating adverse outcomes in subsequent pregnancies, particularly as the number of previous outcomes increases. This latter finding is consistent with the hypothesis of distinct vulnerable newborn phenotypes, which can exhibit varying risks for adverse outcomes based on combinations of prematurity, birth weight, and weight adequacy for gestational age.^35^

Some remarkable findings warrant discussion. First, decreased risk of SGA after long IPIs has been observed in a recent study,^34^ and might be attributed to different risk profile of mothers who wait longer before conceiving again. This group may consist of older women, with a higher prevalence of health issues such as diabetes and obesity, known risk factors for having large-for-gestational-age infants. The increased risks for subsequent LGA associated with longer IPIs, as illustrated in Supplementary Figure S3, reinforce this hypothesis. After restricting the cohort to 2011–2015 births, we found a different risk ratio pattern, with higher increases among women with more prior outcomes. This might be due to differences in index birth outcomes distribution, especially preterm births^36^ (Supplementary Figure S7 and Supplementary Table S4–Supplementary Material), compared to earlier periods, affecting baseline risks in subsequent pregnancies.

Additionally, greater risk ratios of PTB after IPIs <6 months in the group with more previous adverse outcomes contrast with results from a recent publication.^20^ The study showed greater odds ratios (OR) for subsequent PTB after an IPI <6 months among women with a previous term birth compared to those with a previous preterm birth. The lack of information on other birth outcomes (e.g., small-for-gestational age, low birth weight) in the study mentioned could explain the contrast between results. As shown in Supplementary Table S3, women whose index birth occurred on the term but ended with SGA and/or LBW had greater RRs for PTB after short IPIs compared to those with a previous PTB, indicating that even when a child is born full-term, there could be other conditions that pose an additional risk for subsequent preterm delivery.

Moreover, since we couldn't differentiate between spontaneous and indicated preterm births, the observed recurrence tendency in the group with more previous outcomes might be attributed to a higher underlying disease burden. These women had elevated rates of cesarean delivery, more detected congenital anomalies, and inadequate prenatal care during the index pregnancy, possibly related to maternal disease occurrence. Consequently, they could be subjected to more intensive monitoring for complications (e.g., maternal disease decompensation, fetal distress) and an earlier indication of delivery.

Finally, the observed association between IPI and adverse birth outcomes may be confounded by pregnancy intention and postpartum contraception, factors associated with adverse maternal and infant outcomes.^37^ This is of special concern in the context of the Brazilian population, where a significant fraction of pregnancies are unplanned.

In summary, higher baseline risks for subsequent unfavorable outcomes occurred in the group with more previous adverse outcomes. Considering a multiplicative association (risk ratio), the relative impact of IPI is greater when the first pregnancy had no adverse outcomes. Under an additive association (risk difference), the absolute risk increase with changing IPI is higher in those with more prior outcomes.

The findings from this investigation would help policymakers plan interventions on a population basis and practitioners to better advise their patients on birth spacing.

### Strengths and limitations

Our study has several strengths. To our knowledge, this was the largest study investigating the influence of previous outcomes on the association of IPI with subsequent birth outcomes, specifically SGA and LBW. We used a validated technique^26^ that allows highly accurate linkage of nationwide administrative and health databases.

However, some limitations should be considered. Important confounders like contraception methods, pregnancy intention, smoking habits, maternal diseases, and drug use during pregnancy were not available, which may cause bias and limit extrapolation of results. Although some of the variables included in our model, like maternal education, may be on the causal path of many of these confounders, residual confounding remains possible. Moreover, the outcome definitions used in this study, such as SGA, PTB, or LBW, could potentially yield different results if alternative cutoffs or growth charts are applied, and our conclusions may not be applicable in such alternative contexts. In addition, we could not assess other important outcomes such as child and maternal mortality, as these were not available in our dataset.

Although standard approaches are used to estimate gestational ages, different methods could be employed (see Additional Methods–Supplementary Material), potentially resulting in variations in the precision of IPI calculation. Also, our study did not account for the timing of intervening events between pregnancies, which could introduce misclassification of IPIs. However, we evaluated self-reported stillbirth or abortion frequencies occurring between pregnancies (Supplementary Table S5–Supplementary Material), revealing lower rates in shorter IPIs and higher rates in longer IPIs, suggesting a bias primarily among longer IPI groups.

Finally, despite the dataset’s reliability, potential linkage errors may occur, affecting the identification of consecutive events and prior adverse outcomes, resulting in miscalculation of IPI, and introducing bias into the results.

### Conclusions

This large population-based cohort study found that IPIs shorter than 6 months were associated with increased risks for all three outcomes investigated. In contrast, IPIs longer than 120 months were associated with increased risks of PTB and LBW but decreased risks of SGA.

Moreover, our findings demonstrated higher absolute risks of subsequent SGA, LBW, and PTB for women with prior occurrences, irrespective of interpregnancy interval length, indicating increased baseline risks for unfavorable outcomes. Additionally, we observed that the relative influence of changing IPI duration on the risk of subsequent adverse outcomes appears less significant for women with prior outcomes than those without, as evidenced by lower RRs in the former group.

The present findings suggest that previous adverse outcomes may affect the association of IPI and birth outcomes. Clinicians should balance the risks of recurrent pregnancy complications with those following exceedingly short or long IPIs. Further studies are needed to corroborate our results.

## Contributors

JT and EP are the principal investigators and were responsible for data curation and had access and verified all data in the study. EP and MB were responsible for funding acquisition. JT conceived the study, with support from SG, BA, AR, and EP. JT, TS, AR, and EP were responsible for data acquisition. JT and TS performed the statistical analysis that was verified by AR and EP. JT wrote the manuscript, with support from TS, BA, and EP. All authors reviewed and edited the manuscript. TS, LR, MB, AR, RS, IF, and EP supervised research planning and/or execution. All authors have read and approved the final version of the manuscript, and accept responsibility for the decision to submit for publication.

## Data sharing statement

All data supporting the findings presented here were obtained from CIDACS. Importantly, restrictions apply to the availability of these data. However, upon reasonable request and provided all ethical and legal requirements are met, the institutional data curation team can make the data available. Information on how to apply to access the data can be found at https://cidacs.bahia.fiocruz.br/en/.

## Declaration of interests

All authors declare no conflict of interest.
