# Supplementary material for: Association of interpregnancy interval with adverse pregnancy outcomes according to the outcomes of the preceding pregnancy: a longitudinal study with 4.7 million live births from Brazil
Source: Lancet Reg Health Am. 2024 Feb 1;30:100687. doi: 10.1016/j.lana.2024.100687 (PMC10850771; doi:10.1016/j.lana.2024.100687)
Supplement: Supplementary Material [file mmc1.pdf]

SUPPLEMENTARY APPENDIX

Title: Association of Interpregnancy interval with adverse pregnancy outcomes according to the outcomes of the preceding pregnancy: A longitudinal study with 4.7 million live births from Brazil

Authors

João Guilherme G. Tedde, Thiago Cerqueira-Silva, Sidney A. Lagrosa Garcia, Brenda V. Amira, Laura C. Rodrigues, Mauricio L. Barreto, Aline S Rocha, Rita de Cássia Ribeiro-Silva, Ila R Falcão, Enny S. Paixao.

Table of Contents

Additional Methods. 2

Supplementary Figure 1. Illustration of interpregnancy interval (IPI) and time of variable measurements. 3

Supplementary Figure 2. Directed acyclic graph (DAG) with exposure, outcome, confounders, and relationships assumed in our model 4

Supplementary Table 1. Type of index birth outcome according to the number of outcomes (complete cohort – births from 2001-2015) 4

Supplementary Table 2. Predicted absolute risks of subsequent birth outcomes for IPI categories, stratified by number of index birth outcomes 5

Supplementary Figure 3. Risk ratios of large-for-gestational-age (LGA) according to interpregnancy interval and number adverse outcomes at index birth 6

Supplementary Table 3. Risk ratios of subsequent birth outcomes for ipi categories, stratified by the type of index birth outcome (complete cohort – births from 2001-2015) 7

Supplementary Figure 4. Absolute risks of birth outcomes according to interpregnancy interval and number of adverse outcomes at index birth, restricted cohort (2011-2015). 8

Supplementary Figure 5. Risk ratios of birth outcomes according to interpregnancy interval and number of adverse outcomes at index birth, restricted cohort (2011-2015). 9

Supplementary Figure 6. Risk ratios of birth outcomes in the pre-interval (index) birth according to interpregnancy interval: a negative control analysis. 10

Supplementary Figure 7. Prevalence of birth outcomes according to birth year, Brazil, 2001-2015 11

Supplementary Table 4. Type of index birth outcome according to the number of outcomes (restricted cohort - births from 2011 to 2015) 11

Supplementary Table 5. Number of estimated stillbirths or abortions occurring between index and subsequent births, stratified by IPI length 12

R Code for gestational age imputation 13

References 14

Additional Methods.

Data Sources

The Brazilian SINASC system registers live births and is continually updated through the live birth registration process. This mandatory document is completed by a healthcare professional present during the delivery and is organized into eight sections: I - Newborn characteristics; II - Birthplace identification; III - Mother's characteristics; IV - Father's identification; V - Pregnancy and delivery details; VI - Congenital anomalies; VII- identification of the professional completing the notification; VIII- registry office identification. Basically, it contains maternal information (e.g., maternal ethnicity, age, marital status, level of education), pregnancy information (e.g., antenatal visits, gestational age, mode of delivery) and newborn information (e.g., birth weight, sex, presence of congenital anomaly). The forms that provide information to SINASC adopt the last menstrual period (LMP) as the standard method for estimating the gestational age in weeks. Results of physical examinations and other methods are alternatively accepted. Data completeness is very high, with the majority of variables exceeding 90% completion rate and nearly 97% of Brazilian births registered.

The 100 million Brazilian Cohort is primarily built from the Cadastro Único (CadUnico), a tool used by the Brazilian government to evaluate eligibility for more than 20 social programs. To enroll in CadÚnico, a representative within the family must provide information and required documentation for all family members during an interview. This representative should be at least 16 years old and preferably female. The 100 million cohort gathers standardized information for each family member. Data completeness varies by variable, but essential details such as names and municipality of residence are available for all registered individuals. It covers the poorest half of the Brazilian population (families with a monthly income equal to or below three minimum wages [~750 USD]).

Linkage Process

The rate of matched pairs varied across the years. Overall, the percentage of births for which a connection could be established using a defined linkage threshold increased over time, ranging from 39.3% in 2001 to 82.1% in 2014. Also, the initial data cleaning performed for our analysis, in which we could exclude inconsistent information, served to reinforce linkage accuracy in our final dataset.

Further details on data sources and linkage process can be obtained elsewhere[1–5]

Methods for IPI calculation

Interpregnancy interval (IPI) was defined as the time, in months, between the delivery date of the first (index birth) among two consecutive births and the subsequent conception for a given mother. Conception dates were estimated by subtracting gestational ages at birth from delivery dates of the same child. Therefore, IPI was calculated by subtracting the index birth date from the subsequent birth date minus the gestational age at birth of the subsequent pregnancy. Each woman could contribute with up to 4 IPIs. For IPI calculation, only consecutive births were considered (i.e. Women with only pregnancies #1, #2 and #4 recorded in our dataset - supposing pregnancy #3 was lost due to inconsistent information -, would only contribute with 1 IPI (child 1 – child 2). Illustrative example: A woman with 3 consecutive children – child 1, born with 36 weeks of gestational age (GA) in December 15, 2001; child 2, born with 36 weeks of GA in December 15, 2005, and child 3, born also with 36 weeks of GA in December 15, 2010 - would have 2 eligible IPIs. IPI 1 (delivery of child 1 to conception of child 2) and IPI 2 (delivery of child 2 to conception of child 3) would be calculated as:

IPI 1 = (conception date of child 2 - delivery of child 1) → (delivery of child 2 - gestational age of child 2) - delivery of child 1 → (Dec,15,2005 – 36 weeks) - Dec, 15, 2001 ≃ 39 months

IPI 2 = (conception date of child 3 - delivery of child 2) → (delivery of child 3 - gestational age of child 3) - delivery of child 2 → (Dec,15,2010 – 36 weeks) - Dec, 15, 2005 ≃ 51 months

Methods for gestational age imputation for births occurring prior to 2011

Until 2010, gestational age information on SINASC was only recorded as non-overlapping categories of completed weeks, as follows: < 22, 22-27, 28-31, 32-36, 37-41 and ≥ 42 weeks. 2011 onwards, records included also a discrete measure of gestational age in completed weeks (e.g., 28, 32, 34, 37, 40 weeks, …). Therefore, we could not calculate an accurate IPI for the entire cohort. Some considerations guided our decision to adopt an imputation algorithm to gestational age. First, the number of observations with discrete completed weeks (from 2011 to 2015) available in the remaining cohort was large (8,156,006 births), making it possible to extract the distributions of gestational ages. Moreover, we assumed gestational age distributions are unlikely to vary within each category of lengths, making it possible to extend it to preceding years (i.e.,2001-2010). Finally, we considered that the maximum possible deviation in gestational age estimation would still be very small (i.e., imputing 22 weeks when the true gestational age is 27 gives a deviation of 5 weeks, or approximately 1.15 months in births from 22-27 weeks' group) to affect IPI calculation.

The algorithm consisted in extracting frequencies of each gestational age value within its categorical group (e.g., the frequency of 28 week births within all births from the group of 22-31 weeks) in the cohort from 2011 onwards. Finally, we created a function to impute probabilistically (based on the frequencies of occurrence mentioned above) a single discrete gestational age, in completed weeks, to births occurring prior to 2011. The R code employed for imputation is presented at the end of this Supplementary Material (See 'R Code for gestational age imputation' section).

We acknowledged the potential bias associated with the outcome of small-for-gestational age (SGA) due to the variation in weight percentiles used as cutoffs in reference charts based on completed weeks. However, it is unlikely that the imputation process introduced significant bias for preterm birth and low birth weight outcomes, since determination of preterm birth was based on the original gestational age categorical variable (not imputed) available in the SINASC dataset for the entire study period (2001-2015) and birth weight records are not influenced by gestational age value.

Statistical Analysis

Covariates included in primary analysis

Covariates measured at index birth (i.e. the first birth among two consecutive) and adjusted for in the model included: presence of any congenital anomaly (presence or absence), mother's age category (<18, 18-23, 24-29, 30-34, 35-39 and 40-50), number of antenatal visits (0, 1-3, 4-6, ≥ 7), maternal education level in years of study (0, 1-3, 4-7, 8-11, ≥ 12 years), region of birth (South, South, Midwest, North east or North), mode of delivery (vaginal or cesarean), number of live children (0-4) and birth year (2001-2004, 2005-2010, 2011-2015).

Number of adverse outcomes at index birth

The number of adverse outcomes was defined as the cumulative sum of occurrences for any of the following outcomes during the index birth, preceding the IPI: 0 (none), 1 (either small-for-gestational-age, low birth weight, or preterm birth), 2 (combined small-for-gestational-age and low birth weight, or combined preterm birth and low birth weight), or 3 (combined small-for-gestational-age, low birth weight, and preterm birth).

Sensitivity Analyses

To assess the robustness of our results, we conducted three sensitivity analyses. Firstly, we performed a logistic regression model, including all original covariates, but instead of using the cumulative number of previous outcomes, we introduced the type of index birth outcome as a potential effect modifier. This analysis aimed to investigate if interactions varied depending on the specific index birth outcome.

Next, we restricted our analysis to births occurring from 2011 onwards to evaluate the potential bias introduced by our gestational age imputation algorithm. This was achieved by running a logistic regression model that incorporated all outcomes, covariates, and interaction terms used in the primary analysis.

Lastly, we employed the outcome of the index pregnancy as a negative outcome control. Using this approach, we replicated our main analyses while considering the outcome of the first pregnancy as the dependent variable. This methodology was based on the rationale that the interpregnancy interval could not have caused the outcome of the index pregnancy. Any observed association between them would be attributed to residual confounding that persisted over time, thus generating a spurious association.

Supplementary Figure 1. Illustration of interpregnancy interval (IPI) and time of variable measurements.

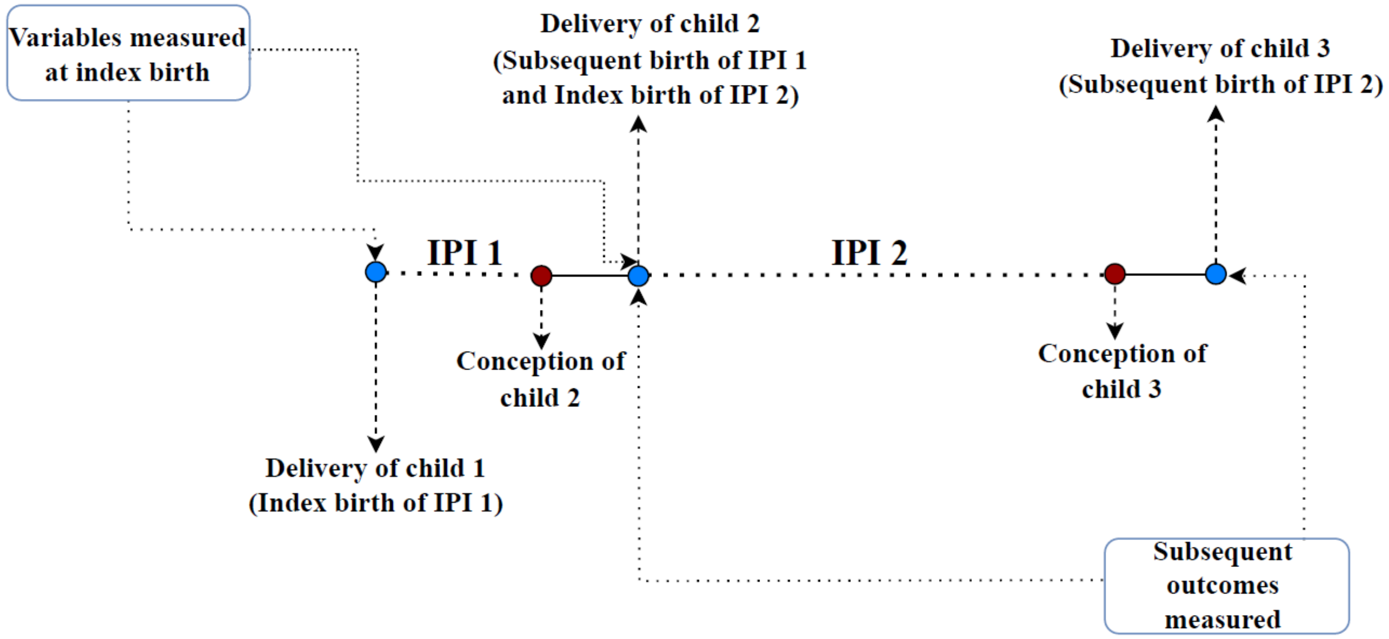

Variables measured at index birth included number of adverse outcomes at the index birth, presence of any congenital anomaly, mother's age, number of antenatal visits, maternal education, region of birth, mode of delivery, number of live children and birth year. Subsequent outcomes included Small for gestational age (SGA), preterm birth (PTB), and low birth weight (LBW).

Supplementary Figure 2. Directed acyclic graph (DAG) with exposure, outcome, confounders, and relationships assumed in our model

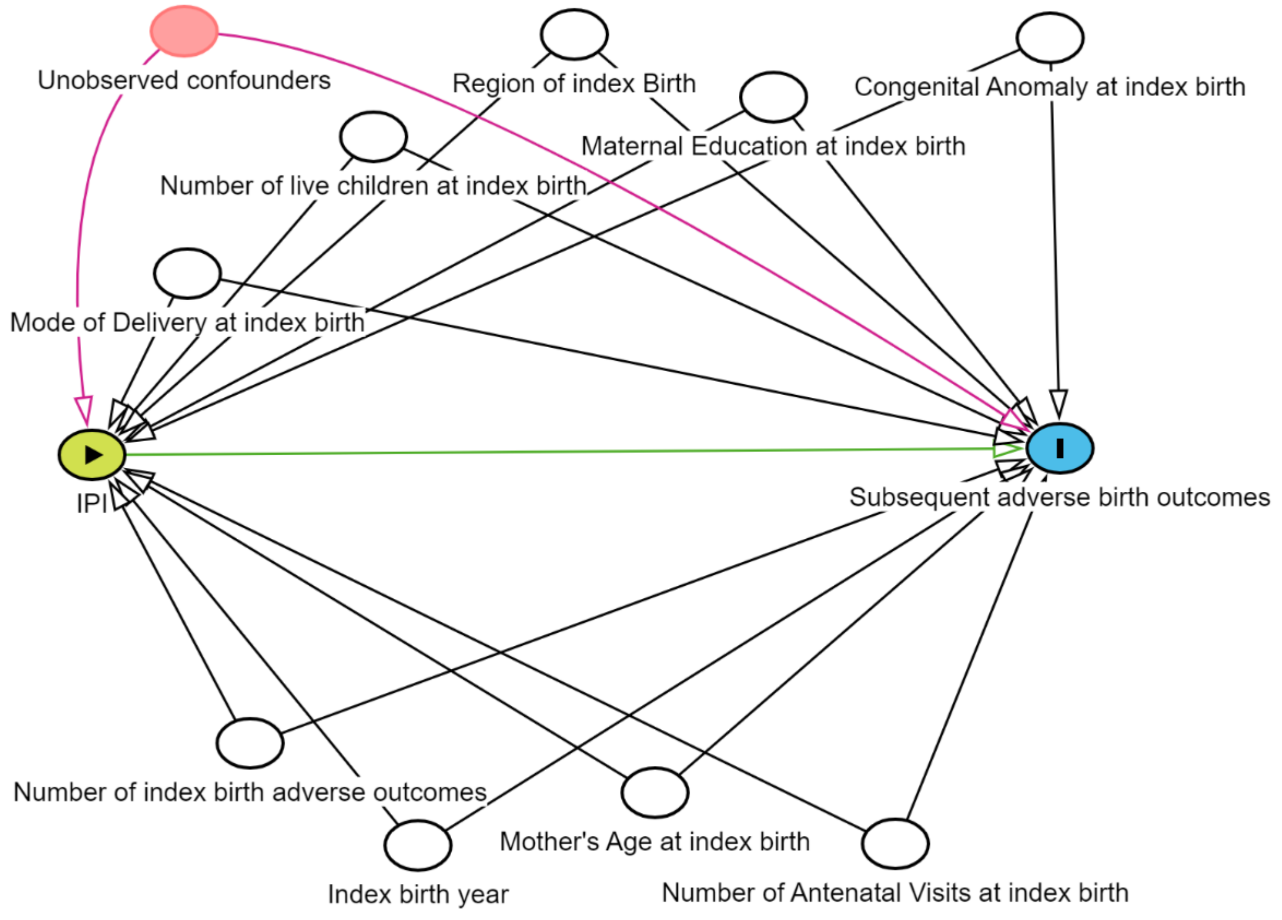

Supplementary Table 1. Type of index birth outcome according to the number of outcomes (complete cohort – births from 2001-2015)

		Number of outcomes at index birth			
**Variables**	**Overall (N = 4,788,279)**	**0 (N = 3,943,890)**	**1 (N = 503,629)**	**2 (N = 298,411)**	**3 (N = 42,349)**
**Type of outcome**					
None	3,943,890 (82.4%)	3,943,890 (100%)	0 (0%)	0 (0%)	0 (0%)
SGA	341,898 (7.1%)	0 (0%)	341,898 (67.9%)	0 (0%)	0 (0%)
PTB	150,641 (3.1%)	0 (0%)	150,641 (29.9%)	0 (0%)	0 (0%)
LBW	11,090 (0.2%)	0 (0%)	11,090 (2.2%)	0 (0%)	0 (0%)
SGA|LBW	180,910 (3.8%)	0 (0%)	0 (0%)	180,910 (60.6%)	0 (0%)
PTB|LBW	117,501 (2.5%)	0 (0%)	0 (0%)	117,501 (39.4%)	0 (0%)
SGA|PTB|LBW	42,349 (0.9%)	0 (0%)	0 (0%)	0 (0%)	42,349 (100%)

Data are presented as n(%)

SGA: Small for gestational age; LBW: Low birth weight; PTB: Preterm birth

Supplementary Table 2. Predicted absolute risks of subsequent birth outcomes for IPI categories, stratified by number of index birth outcomes

	**Interpregnancy interval, adjusted absolute risk % (95% CI)**						
**Outcome**	**<6**	**6-10**	**11-17**	**18-22**	**23-58**	**59-119**	**≥120**
**SGA at subsequent birth**							
Overall	12.0 (11.9-12.1)	9.5 (9.4-9.6)	8.6 (8.5-8.7)	8.2 (8.1-8.3)	7.9 (7.8-8.0)	7.5 (7.4-7.6)	7.2 (7.1-7.4)
Number of outcomes at index birth (N)							
0	9.5 (9.3-9.6)	7.6 (7.5-7.7)	6.9 (6.8-7.0)	6.6 (6.5-6.7)	6.3 (6.2-6.4)	6.0 (5.9-6.0)	5.5 (5.3-5.7)
1	19.8 (19.4-20.3)	16.4 (16.1-16.7)	15.5 (15.2-15.8)	14.6 (14.3-14.9)	14.1 (13.9-14.3)	12.9 (12.7-13.2)	11.0 (10.3-11.7)
2	26.1 (25.5-26.7)	23.8 (23.3-24.2)	23.1 (22.7-23.5)	22.2 (21.7-22.8)	21.4 (21.2-21.7)	18.9 (18.5-19.3)	15.9 (14.8-17.0)
3	27.8 (26.4-29.2)	23.5 (22.3-24.7)	23.6 (22.5-24.6)	23.2 (21.8-24.6)	21.2 (20.5-21.9)	17.8 (16.8-18.7)	16.0 (13.2-18.8)
**Low birth weight at subsequent birth**							
Overall	8.7 (8.6-8.8)	6.4 (6.3-6.5)	5.6 (5.5-5.6)	5.3 (5.2-5.4)	5.4 (5.3-5.4)	6.0 (5.9-6.0)	7.3 (7.2-7.5)
Previous outcomes at index birth (N)							
0	6.0 (5.9-6.1)	4.6 (4.5-4.7)	4.1 (4.0-4.2)	4.0 (3.9-4.1)	4.2 (4.1-4.2)	5.0 (4.8-4.9)	6.4 (6.2-6.6)
1	13.5 (13.1-13.9)	10.9 (10.6-11.2)	9.6 (9.4-9.8)	9.3 (9.1-9.6)	9.8 (9.6-9.9)	11.1 (10.9-11.3)	13.6 (12.8-14.5)
2	27.1 (26.5-27.7)	22.6 (22.1-23.1)	20.8 (20.4-21.2)	19.5 (19.0-20.0)	19.6 (19.3-19.8)	20.2 (19.8-20.6)	22.1 (20.8-23.5)
3	32.5 (31.1-34.0)	27.4 (26.1-28.7)	26.7 (25.6-27.9)	26.2 (24.7-27.7)	25.4 (24.7-26.1)	26.0 (25.0-27.1)	24.1 (20.7-27.6)
**Preterm at subsequent birth**							
Overall	8.4 (8.3-8.5)	7.8 (7.7-7.9)	7.3 (7.2-7.3)	7.1 (7.0-7.2)	7.1 (7.0-7.1)	8.2 (8.1-8.2)	10.7 (10.5-10.9)
Previous outcomes at index birth (N)							
0	6.3 (6.2-6.4)	6.3 (6.2-6.4)	6.1 (6.1-6.2)	6.2 (6.1-6.3)	6.7 (6.7-6.8)	9.0 (8.9-9.1)	15.2 (14.9-15.6)
1	11.9 (11.5-12.2)	11.6 (11.3-11.9)	10.8 (10.6-11.0)	10.8 (10.5-11.1)	10.8 (10.7-11.0)	13.8 (13.5-14.1)	21.0 (19.9-22.2)
2	20.5 (20.0-21.1)	17.9 (17.5-18.3)	16.2 (15.9-16.6)	15.3 (14.8-15.7)	16.5 (16.3-16.8)	20.0 (19.6-20.4)	27.4 (25.8-29.0)
3	25.6 (24.3-27.0)	22.1 (20.9-23.3)	21.3 (20.3-22.4)	21.4 (20.0-22.8)	22.5 (21.8-23.2)	27.1 (26.0-28.2)	35.5 (31.4-39.6)

Data are presented as adjusted absolute risk percentages (lower 95% CI – Upper 95%CI)

IPI: Interpregnancy interval; SGA: Small for gestational age; LBW: Low birth weight; PTB: Preterm birth

Supplementary Figure 3. Risk ratios of large-for-gestational-age (LGA) according to interpregnancy interval and number adverse outcomes at index birth

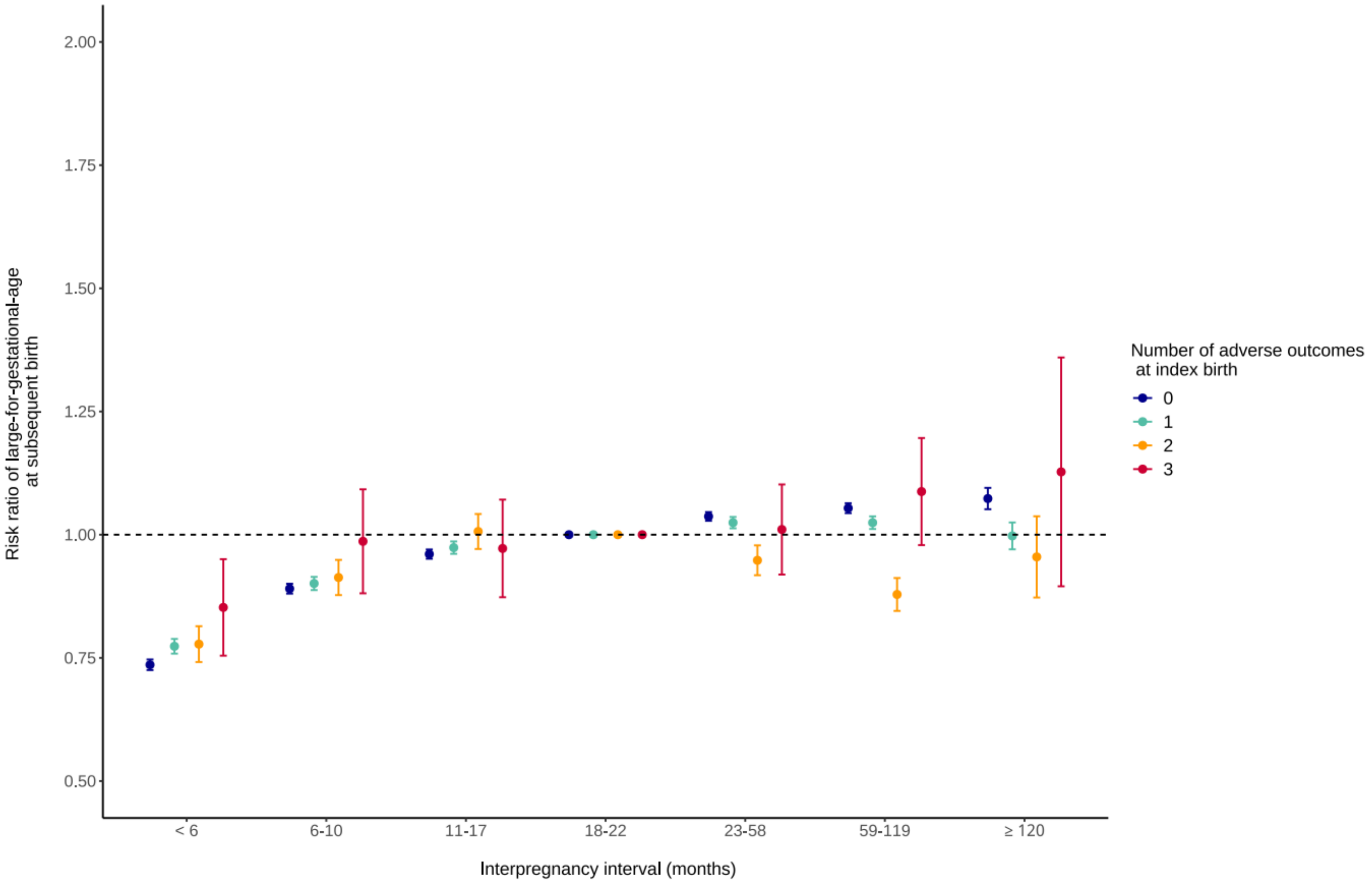

Adjusted risk ratios (95% confidence intervals) of large-for-gestational-age at each interpregnancy interval length and according to the number of outcomes at the index birth. Data refers to years 2001-2015 in 4,422,146 pregnancies.

Supplementary Table 3. Risk ratios of subsequent birth outcomes for IPI categories, stratified by the type of index birth outcome (complete cohort – births from 2001-2015)

	Index birth outcomes - aRR (95%CI)						
**IPI (months)**	**None**	**SGA**	**PTB**	**LBW**	**SGA | LBW**	**PTB | LBW**	**SGA | PTB | LBW**
**SGA at subsequent birth**							
< 6	1.43 (1.40-1.46)	1.34 (1.29-1.39)	1.45 (1.34-1.56)	1.27 (1.06-1.49)	1.25 (1.21-1.30)	1.13 (1.06-1.19)	1.19 (1.10-1.29)
6-10	1.14 (1.12-1.16)	1.13 (1.10-1.17)	1.09 (1.01-1.16)	1.13 (0.96-1.31)	1.08 (1.04-1.12)	1.06 (1.00-1.12)	1.01 (0.92-1.09)
11-17	1.05 (1.03-1.06)	1.07 (1.04-1.11)	1.04 (0.97-1.10)	1.01 (0.86-1.16)	1.04 (1.01-1.08)	1.02 (0.97-1.07)	1.01 (0.93-1.09)
18-22	Ref	Ref	Ref	Ref	Ref	Ref	Ref
23-58	0.96 (0.95-0.97)	0.94 (0.92-0.97)	0.96 (0.90-1.01)	0.98 (0.84-1.11)	0.97 (0.94-1.00)	0.95 (0.90-0.99)	0.91 (0.85-0.98)
59-119	0.91 (0.89-0.92)	0.85 (0.82-0.88)	0.87 (0.81-0.93)	0.84 (0.70-0.98)	0.87 (0.84-0.90)	0.81 (0.77-0.86)	0.77 (0.70-0.83)
≥120	0.84 (0.81-0.87)	0.70 (0.64-0.75)	0.86 (0.71-1.01)	1.00 (0.66-1.35)	0.73 (0.66-0.79)	0.68 (0.58-0.78)	0.69 (0.56-0.82)
**Low birth weight at subsequent birth**							
< 6	1.49 (1.45-1.52)	1.37 (1.31-1.44)	1.63 (1.50-1.76)	1.32 (1.07-1.57)	1.33 (1.27-1.39)	1.43 (1.35-1.50)	1.24 (1.15-1.33)
6-10	1.15 (1.12-1.17)	1.14 (1.08-1.19)	1.24 (1.14-1.33)	1.09 (0.90-1.28)	1.10 (1.05-1.15)	1.22 (1.16-1.28)	1.05 (0.97-1.13)
11-17	1.02(1.00-1.04)	1.02 (0.97-1.06)	1.08 (1.00-1.16)	0.78 (0.64-0.92)	1.04 (1.00-1.08)	1.10 (1.05-1.15)	1.02 (0.94-1.09)
18-22	Ref	Ref	Ref	Ref	Ref	Ref	Ref
23-58	1.04 (1.02-1.06)	1.00 (0.96-1.04)	1.12 (1.05-1.19)	0.92 (0.78-1.06)	1.00 (0.97-1.04)	1.00 (0.96-1.04)	0.97 (0.90-1.03)
59-119	1.22 (1.19-1.24)	1.13 (1.09-1.18)	1.29 (1.19-1.39)	0.88 (0.72-1.04)	1.06 (1.02-1.10)	1.00 (0.95-1.05)	1.00 (0.94-1.07)
≥120	1.59 (1.53-1.65)	1.33 (1.23-1.44)	1.83 (1.55-2.10)	1.24 (0.79-1.68)	1.16 (1.06-1.26)	1.10 (0.98-1.22)	0.92 (0.77-1.06)
**Preterm at subsequent birth**							
< 6	1.02 (0.99-1.04)	1.10 (1.04-1.17)	1.07 (1.01-1.13)	1.19 (0.93-1.46)	1.18 (1.10-1.25)	1.34 (1.28-1.41)	1.19 (1.09-1.29)
6-10	1.00 (0.99-1.02)	1.05 (0.99-1.10)	1.08 (1.02-1.13)	1.17 (0.94-1.41)	1.07 (1.01-1.13)	1.20 (1.15-1.26)	1.03 (0.94-1.12)
11-17	0.98 (0.97-1.00)	0.97 (0.93-1.02)	1.02 (0.97-1.07)	0.90 (0.72-1.08)	0.99 (0.94-1.04)	1.11 (1.06-1.16)	0.99 (0.91-1.07)
18-22	Ref	Ref	Ref	Ref	Ref	Ref	Ref
23-58	1.07 (1.06-1.09)	1.05 (1.01-1.10)	0.98 (0.94-1.03)	0.92 (0.76-1.09)	1.08 (1.03-1.14)	1.07 (1.03-1.12)	1.04 (0.97-1.12)
59-119	1.43 (1.41-1.45)	1.40 (1.34-1.46)	1.22 (1.15-1.28)	1.19 (0.95-1.43)	1.39 (1.32-1.46)	1.23 (1.17-1.29)	1.25 (1.16-1.35)
≥120	2.42 (2.36-2.49)	2.26 (2.10-2.43)	1.64 (1.45-1.82)	1.44 (0.79-2.09)	1.94 (1.76-2.12)	1.66 (1.51-1.81)	1.65 (1.43-1.87)

Data are presented as risk ratios (lower 95% CI – Upper 95%CI)
IPI: Interpregnancy interval; SGA: Small for gestational age; LBW: Low birth weight; PTB: Preterm birth

Supplementary Figure 4. Absolute risks of birth outcomes according to interpregnancy interval and number of adverse outcomes at index birth, restricted cohort (2011-2015).

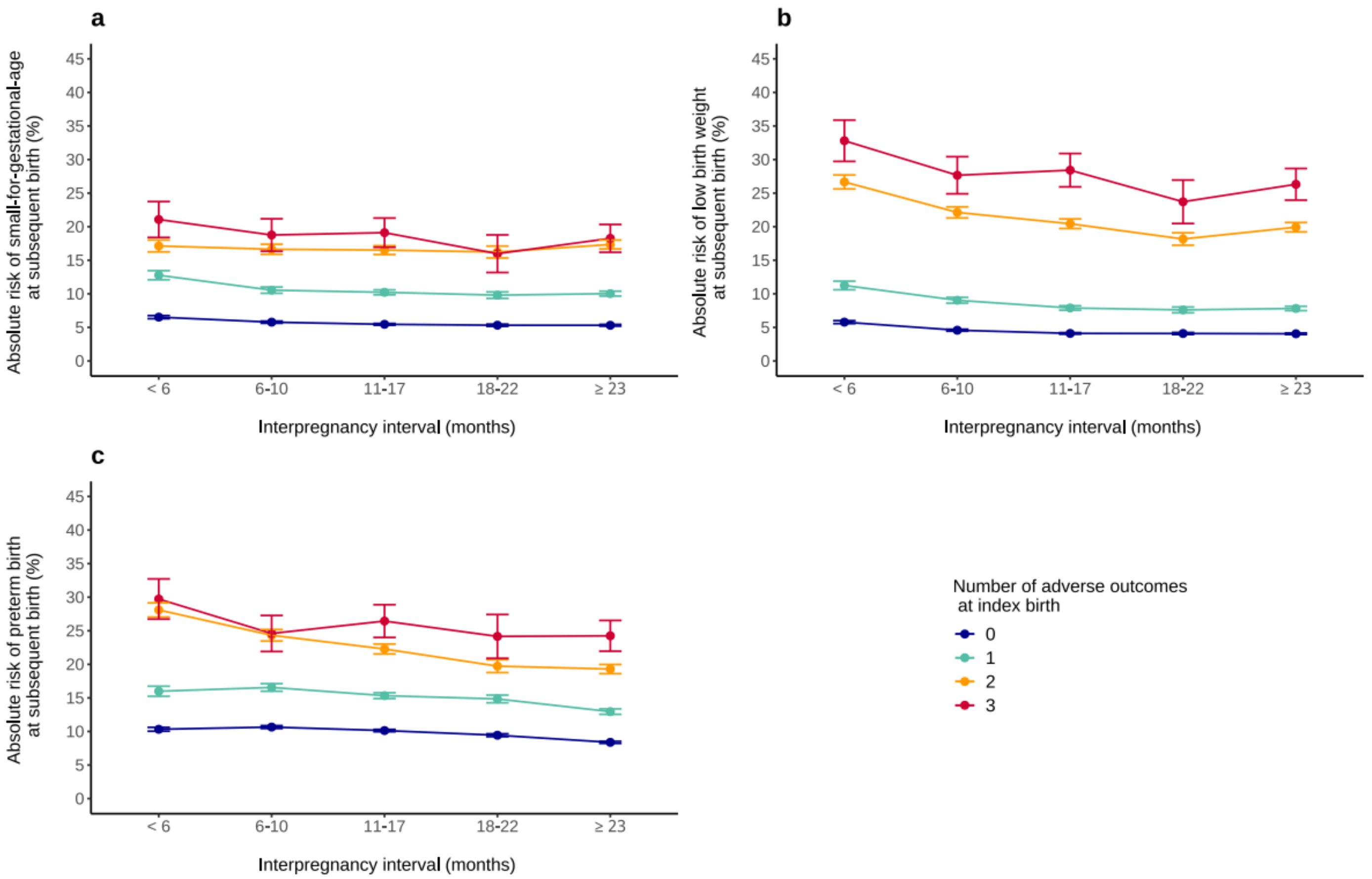

Adjusted absolute risks (95% confidence intervals) of small-for-gestational-age (a), low birth weight (b) and preterm birth (c) at subsequent birth for each interpregnancy interval length and according to the number of outcomes at the index birth. Data refers to years 2011-2015 in 681,236 Pregnancies

Supplementary Figure 5. Risk ratios of birth outcomes according to interpregnancy interval and number of adverse outcomes at index birth, restricted cohort (2011-2015).

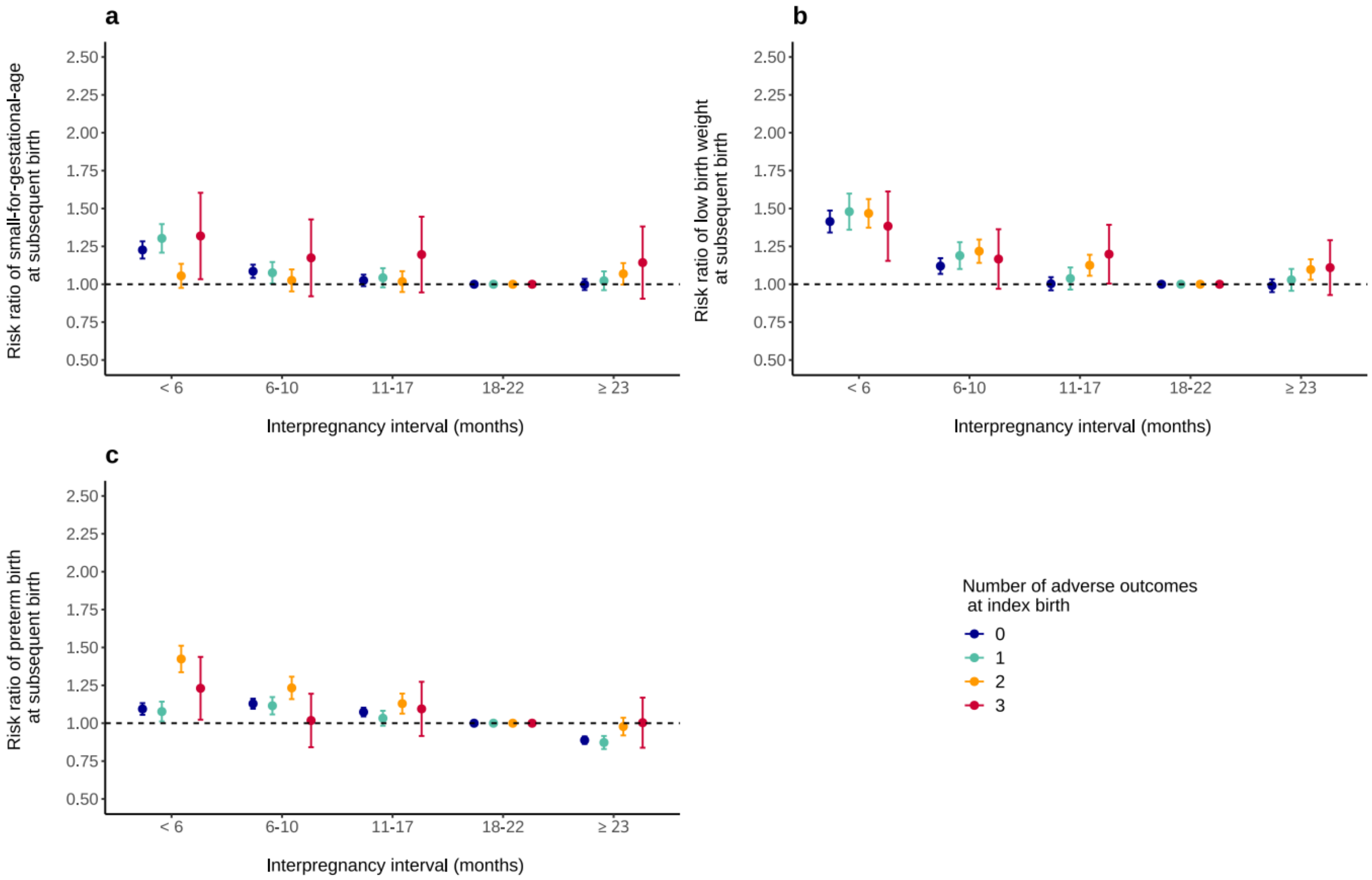

Adjusted risk ratios (95% confidence intervals) of small-for-gestational-age (a), low birth weight (b) and preterm birth (c) at subsequent birth for each interpregnancy interval length and according to the number of outcomes at the index birth. Data refers to years 2011-2015 in 681,236 pregnancies

Supplementary Figure 6. Risk ratios of birth outcomes in the pre-interval (index) birth according to interpregnancy interval: a negative control analysis.

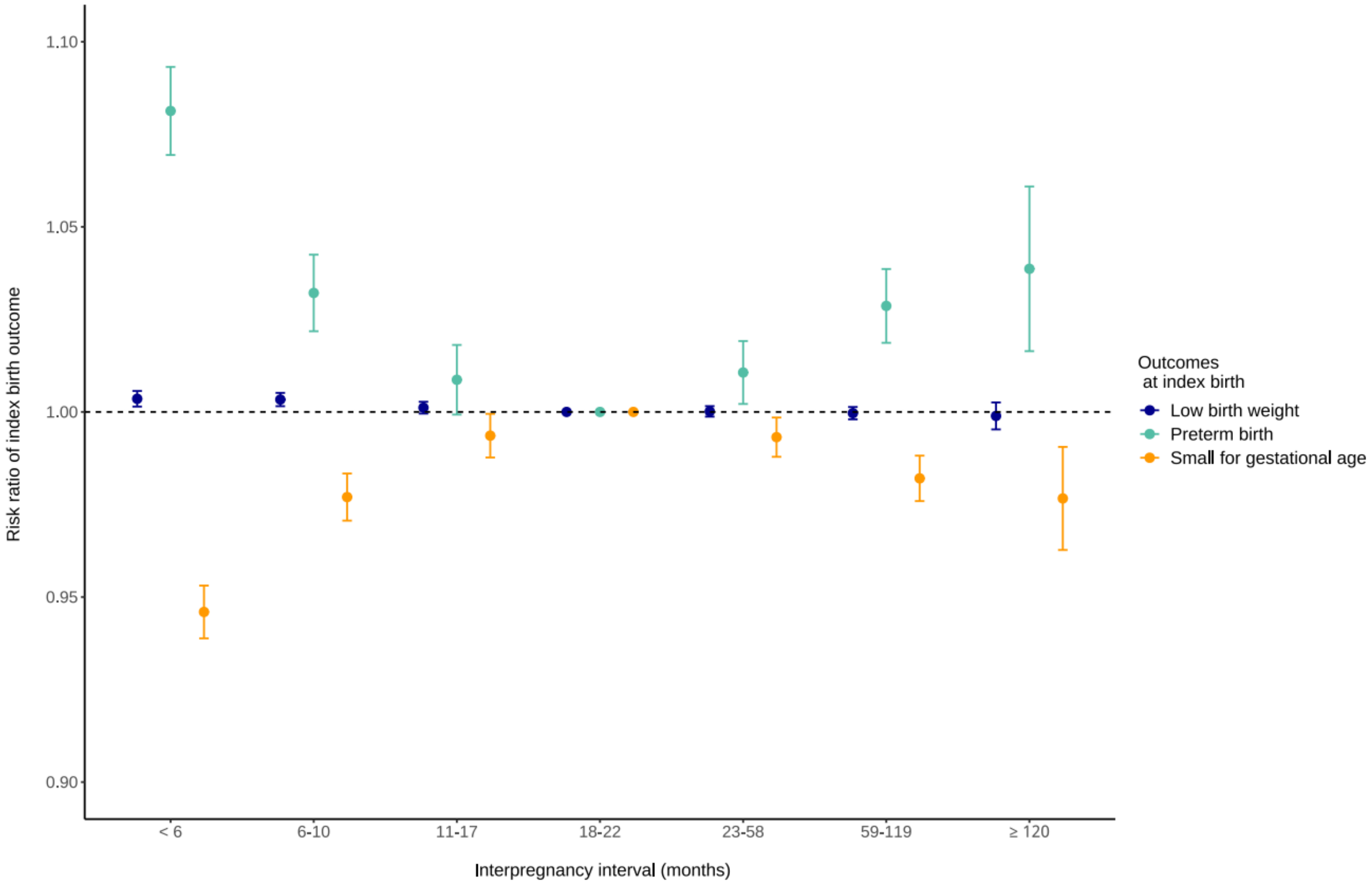

Adjusted risk ratios (95% confidence intervals) of small-for-gestational-age, low birth weight and preterm birth in the pre-interval (index) birth according to interpregnancy interval. Data refers to years 2001-2015 in 4,422,146 pregnancies

Supplementary Figure 7. Prevalence of birth outcomes according to birth year, Brazil, 2001-2015

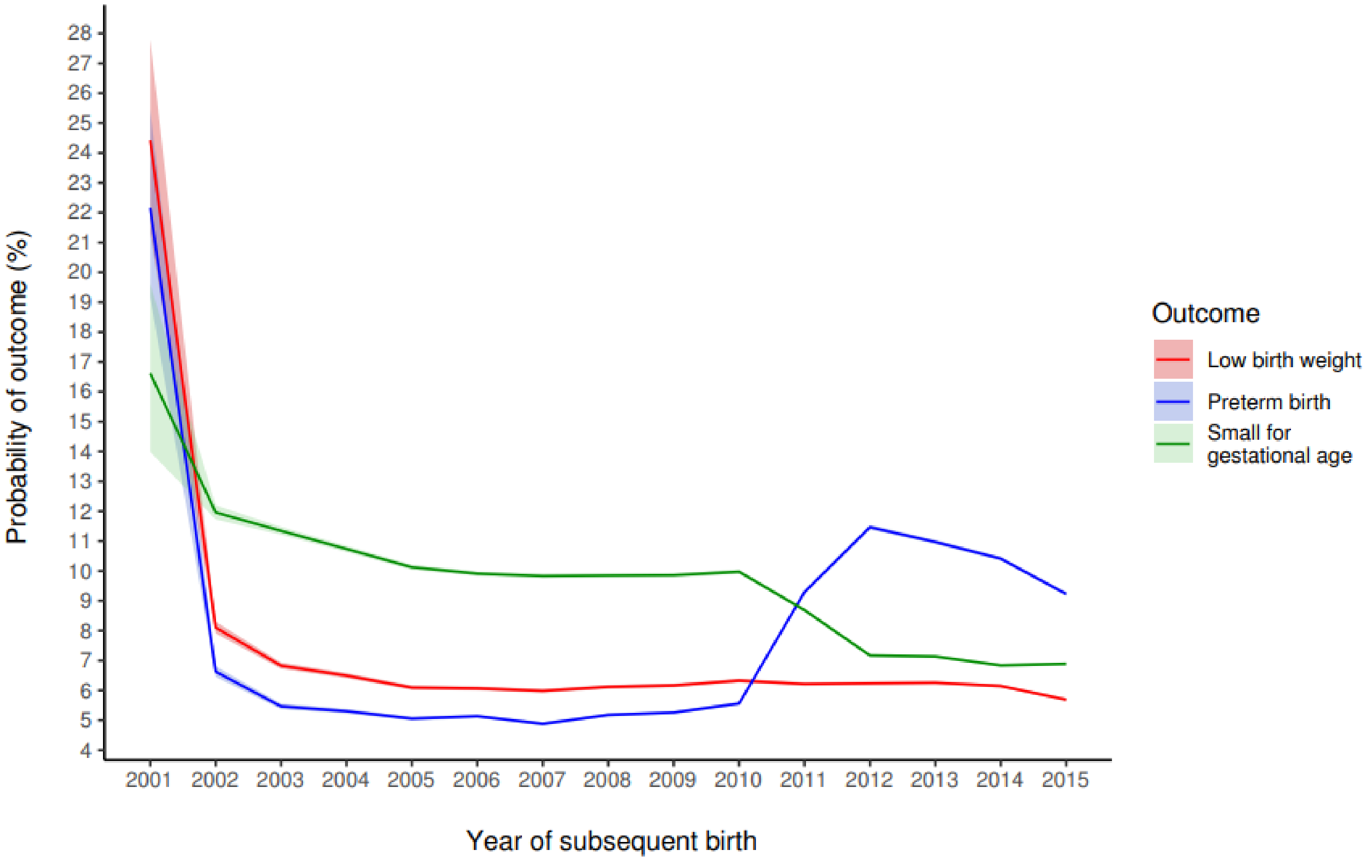

Prevalence and 95% confidence intervals of each birth outcome across the study period. Data refers to 4,788,279 pregnancies

Supplementary Table 4. Type of index birth outcome according to the number of outcomes (restricted cohort - births from 2011 to 2015)

Variables	Overall (N = 714,061)	Number of outcomes at index birth 0 (N = 556,020)	1 (N = 100,838)	2 (N = 51,746)	3 (N = 5,457)
**Type of outcome**					
None	556,020 (77.9%)	556,020 (100%)	0 (0%)	0 (0%)	0 (0%)
SGA	41,947 (5.9%)	0 (0%)	41,947 (41.6%)	0 (0%)	0 (0%)
PTB	56,069 (7.9%)	0 (0%)	56,069 (55.6%)	0 (0%)	0 (0%)
LBW	2,822 (0.4%)	0 (0%)	2,822 (2.8%)	0 (0%)	0 (0%)
SGA|LBW	24,313 (3.4%)	0 (0%)	0 (0%)	24,313 (47.0%)	0 (0%)
PTB|LBW	27,433 (3.8%)	0 (0%)	0 (0%)	27,433 (53.0%)	0 (0%)
SGA|PTB|LBW	42,349 (0.8%)	0 (0%)	0 (0%)	0 (0%)	5,457 (100%)

Data are presented as n(%)

SGA: Small for gestational age; LBW: Low birth weight; PTB: Preterm birth

Supplementary Table 5. Number of estimated stillbirths or abortions occurring between index and subsequent births, stratified by IPI length

IPI	Events*	N available**	Events (%)
< 6	10,558	213,261	4.9
6-10	20,500	380,490	5.4
11-17	39,047	607,033	6.4
18-22	29,290	371,906	7.9
23-58	173,789	1,525,709	11.4
59-119	125,051	721,518	17.3
≥120	15,766	64,787	24.3
**Total**	**414,001**	**3,884,704**	**10.6**

Data was obtained from maternal self-report at the time of delivery for both the index and subsequent births. We compared the reported numbers from both instances to assess the occurrence of stillbirths or abortions between the two pregnancies.
* Absolute number of stillbirths or abortions
**After excluding observations with inconsistent or missing information for number of fetal losses or abortions, the total number of IPIs available for stillbirth estimation was 3,884,704 (81.1%)
IPI: Interpregnancy interval

R Code for gestational age imputation

```
# Hypothetical data frame representing information from prior to 2011.

#This dataset includes a variable "gestacao_sinasc" indicating the corresponding gestational age category for each birth.

#if "gestacao_sinasc" == 2, then "gestational_age_weeks" == "22 to 27 weeks"

#if "gestacao_sinasc" == 3, then "gestational_age_weeks" == "28 to 31 weeks"

#if "gestacao_sinasc" ==  4, then "gestational_age_weeks" == "32 to 36 weeks"

#if "gestacao_sinasc" ==  5, then "gestational_age_weeks" == "37 to 41 weeks"

#if "gestacao_sinasc" == 6, then "gestational_age_weeks" == "42 to 45 weeks"

df <- data.frame(gestacao_sinasc = sample(2:6, size = 120, replace = T)) %>% mutate(

  gestational_age_weeks = case_when(

                gestacao_sinasc == 2 ~ "22 to 27 weeks",

                 gestacao_sinasc == 3 ~ "28 to 31 weeks",

                 gestacao_sinasc == 4 ~ "32 to 36 weeks",

                 gestacao_sinasc == 5 ~ "37 to 41 weeks",

                 gestacao_sinasc == 6 ~ "42 to 45 weeks",

                 TRUE ~ NA_character_))

#Function for estimating discrete gestational ages (GAs) in data before 2011 using observed frequency distributions of

#discrete GAs from the dataset starting in 2011. The imputation was based on frequencies observed within groups of
"gestacao_sinasc"

#at the 2011 onwards dataset.

#The values inside "prob" argument are the observed frequencies. For example, in the 2011-2015 dataset,

#the observed proportions of gestational ages of 22, 23, 24, 25, 26, and 27 weeks within the group category 2 were

#9%, 12%, 16%, 17%, 22%, and 24%, respectively.

#'Impute IG

#'

#'@param group = gestational age category (2: 22 to 27 weeks; 3: 28 to 31 weeks;

#' 4: 32 to 36 weeks; 5: 37 to 41 weeks; 6: 42 to 45 weeks)

#'

#'@param n = number of values to impute

#'
```

```
#'@return imputed values vector

#'@export

#'

#'@examples Must have rowwise() and mutate preceding it. (e.g.,dataframe %>% rowwise() %>% mutate(xx = impute_ig()))

impute_ig <- Vectorize(function(group = gestacao_sinasc,n = 1) {

  data.table::fcase(

    group == 2,sample(x = c(22,23,24,25,26,27), n, replace = T, prob = c(0.09,0.12,0.16,0.17,0.22,0.24)),

    group == 3,sample(x = c(28,29,30,31), n, replace = T, prob = c(0.17,0.20,0.26,0.37)),

    group == 4,sample(x = c(32,33,34,35,36), n, replace = T, prob = c(0.08,0.1,0.17,0.26,0.39)),

    group == 5,sample(x = c(37,38,39,40,41), n, replace = T, prob = c(0.1,0.21,0.32,0.26,0.11)),

    group == 6,sample(x = c(42,43,44,45), n, replace = T, prob = c(0.6,0.22,0.11,0.07))

  )

})

#Imputing gestational ages

df <- df %>% rowwise() %>% mutate(imputed_gestational_age = impute_ig(group = gestacao_sinasc, n = 1))

#Observing results

view(df)
```

References

1. Oliveira MM de, Andrade SSC de A, Dimech GS, et al. Avaliação do Sistema de Informações sobre Nascidos Vivos. Brasil, 2006 a 2010. *Epidemiol Serv Saúde*. 2015;24:629-640. doi:10.5123/S1679-49742015000400005

2. Almeida D, Gorender D, Ichihara MY, et al. Examining the quality of record linkage process using nationwide Brazilian administrative databases to build a large birth cohort. *BMC Med Inform Decis Mak*. 2020;20(1):173. doi:10.1186/s12911-020-01192-0

3. Barreto ML, Ichihara MY, Pescarini JM, et al. Cohort Profile: The 100 Million Brazilian Cohort. *International Journal of Epidemiology*. 2022;51(2):e27-e38. doi:10.1093/ije/dyab213

4. Paixao ES, Cardim LL, Falcao IR, et al. Cohort Profile: Centro de Integração de Dados e Conhecimentos para Saúde (CIDACS) Birth Cohort. *International Journal of Epidemiology*. 2021;50(1):37-38. doi:10.1093/ije/dyaa255

5. Brazilian Ministry of Health. Instruction manual to complete the Live Birth Declaration. 2022;(4). Accessed January 20, 2023. https://bvsms.saude.gov.br/bvs/publicacoes/declaracao_nascido_vivo_manual_4ed.pdf
